# Efficient breast cancer mammograms diagnosis using three deep neural networks and term variance

**DOI:** 10.1038/s41598-023-29875-4

**Published:** 2023-02-15

**Authors:** Ahmed S. Elkorany, Zeinab F. Elsharkawy

**Affiliations:** 1 Department of Electronics and Electrical Comm. Eng., Faculty of Electronic Engineering, Menoufia University, Menouf, 32952 Egypt; 2 Engineering Department, Nuclear Research Center, Egyptian Atomic Energy Authority, Cairo, Egypt

**Keywords:** Cancer, Computer science, Biomedical engineering

## Abstract

Breast cancer (BC) is spreading more and more every day. Therefore, a patient's life can be saved by its early discovery. Mammography is frequently used to diagnose BC. The classification of mammography region of interest (ROI) patches (i.e., normal, malignant, or benign) is the most crucial phase in this process since it helps medical professionals to identify BC. In this paper, a hybrid technique that carries out a quick and precise classification that is appropriate for the BC diagnosis system is proposed and tested. Three different Deep Learning (DL) Convolution Neural Network (CNN) models—namely, Inception-V3, ResNet50, and AlexNet—are used in the current study as feature extractors. To extract useful features from each CNN model, our suggested method uses the Term Variance (TV) feature selection algorithm. The TV-selected features from each CNN model are combined and a further selection is performed to obtain the most useful features which are sent later to the multiclass support vector machine (MSVM) classifier. The Mammographic Image Analysis Society (MIAS) image database was used to test the effectiveness of the suggested method for classification. The mammogram's ROI is retrieved, and image patches are assigned to it. Based on the results of testing several TV feature subsets, the 600-feature subset with the highest classification performance was discovered. Higher classification accuracy (CA) is attained when compared to previously published work. The average CA for 70% of training is 97.81%, for 80% of training, it is 98%, and for 90% of training, it reaches its optimal value. Finally, the ablation analysis is performed to emphasize the role of the proposed network’s key parameters.

## Introduction

In every country (i.e., rich or developing) in the world, women can develop BC at any age after puberty, however, the incidence rates rise as people age^1^. BC is still the second most common cancer in the world and is still fatal to women^2^. BC is a condition in which the breast's cells grow abnormally. Both men and women can develop BC, but women are much more likely to do so. There are three basic components of a breast: connective tissue, ducts, and lobules. Blood and lymph vessels are two ways that BC can travel outside of the breast. BC is said to have metastasized when it spreads to other body regions. The malignant development is initially restricted to the duct or lobule, where it often exhibits no symptoms and has a low risk of spreading^1^. These tumors may develop over time and spread to neighboring lymph nodes or other body organs after invading the breast tissue around them. Widespread metastases are the cause of breast cancer deaths in women. Treatment for breast cancer can be quite successful, especially if the disease is discovered early. The likelihood of surviving BC is increased by routine screening.

Various imaging modalities have been created and used for image acquisition over time. DL approaches have been applied to medical imaging data, including X-ray and magnetic resonance imaging (MRI) images, demonstrating their effectiveness in identifying and tracking illnesses^2–7^. To assess the usefulness of various imaging modalities, standard metrics like sensitivity and specificity are also provided. Mammograms are the primary topic of the research^3,4^.

Digital mammogram analysis using mammography is a reliable early detection technique^2–7^. BC comes in a wide variety of forms, making classification challenging^8^. The kind of BC is determined by which breast cells develop into cancer. The most efficient therapy approach is made possible by the precise classification of the kind of BC. Since human classification is not always exact, an automated accurate breast cancer diagnosis may be advantageous.

Several techniques had been used to classify BC using the MIAS database^9^, such as Bayesian Neural Networks^10^, Relevance Feedback (RF) and Relevance Feedback extreme learning machine (RF-ELM)^11^, optimized kernel extreme learning machine (KELM)^12^, K-nearest neighbor (KNN)^13^, Discrimination Potentiality (DP)^14^, SVM^15–17^, and DL CNN^18^. DL, a component of machine learning algorithms, is primarily focused on automatically extracting and classifying image features. As a result, DL is now a fundamental component of automated clinical decision-making^4,18^. Residual Neural Network (ResNet)^19–21^, Inception-V3^20^, ShuffleNet^22^, Squeeznet^22^, DenseNet^23^, GoogleNet^21,24^, AlexNet^21,24,25^, VGG^21^, and Xception^26^ are some of the most practical DL algorithms that have lately demonstrated the best performance for a variety of machine learning systems.

This work's goal is to offer a precise automated BC classification method using deeply learned features of three different CNN architectures and a TV algorithm as a feature selector to obtain the images’ important features, hence improving the CA. The TV algorithm, which has previously been used in feature selection for text mining and clustering^27,28^, has never been used in BC diagnosis applications. The proposed approach attempts to combine features from the ResNet50, InceptionV3, and AlexNet architectures. The TV model is then used to decrease the number of features by picking the ones with the highest rankings. This increases the classification accuracy and results in a more efficient BC diagnosis system. The suggested system results outperformed previously published findings using the same BC image dataset.

The current proposed study involves the following stages.

- *Patches of interest (i.e., ROI)*: Instead of using whole images, patches are employed to optimize our analysis. It aids in improved performance in addition to efficient computing. From the 322 images in MIAS, 416 image patches have been extracted.
- *Feature extraction:* To extract features, a hybrid model employs three different pretrained DL architectures, namely ResNet50, Inception-V3, and AlexNet.
- *TV Feature selection:* For the first time, TV is employed as a feature selector, selecting the appropriate features from the combined features of the BC image patches.
- *Classification process:* The TV-selected features are used to train and test the MSVM classifier.

The rest of the paper is structured as follows: The related work is outlined in “Related work” section. The methods employed in the suggested strategy are discussed in “The methodology” section. The suggested method's experimental setup is shown in “Experimental setup” section. The proposed CNNs + TV + SVM results are shown and discussed in “Results and discussion” section. “Conclusion” section summarizes the conclusion.

## Related work

Recently, numerous studies using publicly available MIAS mammography images for BC diagnosis and classification have been proposed in the literature. In the last ten years, several computer-aided CAD diagnosis models have been presented for classifying digital mammograms based on three crucial concepts: feature extraction, feature reduction, and image classification. Several researchers have put forth several feature extraction strategies, with improvements made in the detection and classification portions^4,5^.

A Medical Active leaRning and Retrieval (MARRow) method was put forth in^29^ as a means of assisting BC detection. This technique, which is based on varying degrees of diversity and uncertainty, is dedicated to the relevance feedback (RF) paradigm in the content-based image retrieval (CBIR) process. A precision of 87.3% was attained. An automated mass detection algorithm based on Gestalt psychology was presented by Wang et al.^30^. Sensation and semantic integration, and validation are its three modules. This approach blends aspects of human cognition and the visual features of breast masses. Using 257 images, a sensitivity of 92% was reached. In^31^, a hybrid CAD framework was proposed for Mammogram classification. This framework contains four modules: ROI generation using cropping operation, texture feature extraction using contourlet transformation, a forest optimization algorithm (FOA) to select features, and classifiers like k-NN, SVM, C4.5, and Naive Bayes for classification.

In^32^, an efficient technique for ambiguous area detection in digital mammograms was introduced. This technique depends on Electromagnetism-like Optimization (EML) for image segmentation after the 2D Median noise filtering step. The SVM classifier receives the extracted feature for classification. With just 56 images, an accuracy of 78.57% was achieved. By combining deep CNN (DCNN) and SVM, a CAD system for breast mammography has been presented in^33^. SVM was used for classification, and DCNN was employed to extract features. This system achieved accuracy, sensitivity, and specificity of 92.85, 93.25, and 90.56% respectively.

In^34^, CNN Improvement for BC Classification (CNNI-BCC) algorithm was proposed. This method improves the BC lesion classification for benign, malignant, and healthy patients with 89.47% of sensitivity and an accuracy of 90.5%. Hassan et al. presented an automated algorithm for BC mass detection depending on the feature matching of different areas utilizing Maximally Stable Extremal Regions (MSER)^35^. The system was evaluated using 85 MIAS images, and it was 96.47% accurate in identifying the locations of masses. Patil et al. introduced an automated BC detection method^36^, depending on a combination of recurrent neural network (RNN) and CNN. The Firefly updated chicken-based CSO (FC-CSO) was used to increase segmentation accuracy and optimize the combination of RNN and CNN. A 90.6% accuracy, a 90.42% sensitivity, and an 89.88% specificity are obtained. In^37^, a BC classification method named BDR-CNN-GCN was introduced, the is a combination of dropout (DO), batch normalization (BN), and two advanced NN (CNN, and graph convolutional network (GCN)). On the breast MIAS dataset, the BDR-CNN-GCN algorithm was run ten times, yielding 96.00% specificity, 96.20% sensitivity, and 96.10% accuracy.

For the early diagnosis of BC, Shen et al. introduced a CAD system^38^. To extract features, GLCM is combined with discrete wavelet decomposition (DWD), and Deep Belief Network (DBN) is utilized for classification. To enhance DBN CA, the sunflower optimization technique was applied. The findings demonstrated that the suggested model achieves accuracy, specificity, and sensitivity rates of 91.5%, 72.4%, and 94.1%, respectively. In^39^, an automated DL-based BC diagnosis (ADL-BCD) algorithm was introduced utilizing mammograms. The feature extraction step used the pretrained ResNet34, and its parameters were optimized using the chimp optimization algorithm (COA). The classification stage was then performed using a wavelet neural network (WNN). For 70% training and 90% training, the average accuracy was 93.17% and 96.07%, respectively.

In^6^, a CNN ensemble model based on transfer learning (TL) was introduced to classify benign and malignant cancers in breast mammograms. In order to improve prediction performance, the pre-trained CNNs (VGG-16, ResNet-50, and EfficientnetB7) were integrated depending on TL. The findings revealed a 99.62% accuracy, 99.5% precision, 99.5% specificity, and 99.62% sensitivity.

A CNN model was developed by Muduli et al. to distinguish between benign and malignant BC mammography images^40^. Only one fully connected layer and four convolutional layers make up the model's five learnable layers. The findings revealed a 96.55% accuracy in distinguishing between benign and malignant tumors. Alruwaili et al. presented an automated algorithm based on TL for BC identification^41^. Utilizing ResNet50 for evaluation, the model had an accuracy of 89.5%, while using the Nasnet-Mobile network, it had an accuracy of 70%. The transferable texture CNN (TTCNN) is introduced in^42^ for improving BC categorization. Deep features were recovered from eight DCNN models that were fused, and robust characteristics were chosen to distinguish between benign and malignant breast tumors. The results showed a sensitivity of 96.11%, a specificity of 97.03%, and an accuracy of 96.57%.

Oza et al.^5^ provide a review of the image analysis techniques for mammography questionable region detection. This paper examines many scientific approaches and methods for identifying questionable areas in mammograms, ranging from those based on low-level image features to the most recent algorithms. Scientific research shows that the size of the training set has a significant impact on the performance of deep learning methods. As a result, many deep learning models are susceptible to overfitting and are unable to create output that can be generalized. Data augmentation is one of the most prominent solutions to this issue^7^.

According to empirical analysis, when it comes to the training-test ratio, the best results are obtained when 70–90% of the initial data are used for training and the rest are used for testing^43,44^. In addition, 70%, 80%, and 90% dataset splitting ratios are most frequently used for training, as seen in^12,13,18,23,31,39^, and^16,30,39,41^, respectively.

Considering this, it can be said that numerous researchers have examined BC detection and classification and have put up various solutions to this issue. However, the majority of them fell short of the necessary high accuracy, particularly for cases belonging to the three classes of benign, malignant, and healthy cases. As a result, the proposed study aims to improve the automatic classification of breast mammography patches as normal, benign, or malignant. This is possible by combining features from three separate pretrained architectural deep learning networks. The robust high-ranking features are then extracted using the TV feature selection approach. They fed the MSVM classifier to finish the classification task.

## The methodology

The goal of this work was to enhance a mammogram-based BC diagnosis model employing 3-class cases. Following is a detailed explanation of the prepared dataset and the suggested methodology.

### Dataset

The MIAS created and provided the applied digital mammography datasets, which are widely utilized and freely accessible online for research. The images dataset was introduced in Portable Gray Map (PGM) image format. Each mammography in a Mini-MIAS image has a left- and right-oriented breast and is classified as normal, benign, or malignant. Three different forms of breast background tissue are shown in this collection of images: fatty (F), dense-glandular (D), and fatty-glandular (G). The radiologists' ground truth estimates of the abnormality's center and a rough estimate of the circle's radius enclosing the abnormality. This indicates where the lesion is, so we do a cropping operation on the mammograms that were taken from the standard dataset to extract the ROI of any abnormal area. Mammogram abnormalities or ROIs are extracted and labeled as image patches. For normal mammograms, the ROI is randomly chosen. Table 1 contains a list of the segregated ROI image patches.

**Table 1:** Mammogram image patches distribution of MIAS dataset.

	Normal	Benign	Malignant	Total
D	100	23	17	140
F	100	23	17	140
G	100	20	16	136
All	300	66	50	416

### The proposed approach

A method for automatically detecting and categorizing BC in mammograms based on deeply learned features is suggested. The pretrained feature extraction models i.e., ResNet50, AlexNet, and Inception-V3 are hired. ResNet is a 50-layer neural network trained on the ImageNet dataset. It creates shortcuts between layers to avoid distortion as the network grows deeper and more complicated. AlexNet is a type of CNN that has gained worldwide recognition. It has five convolution layers, pooling layers, and three fully connected (FC) layers. Inception-V3 was created with DL techniques to aid in object detection and image analysis. It has 48 deep layers trained on the ImageNet dataset, including convolution, maximum pooling, and FC layers. The TV feature selection algorithm is then used to pick the most reliable features. The MSVM is employed to perform the classification task. Table 2 lists the introduced network parameters where all DL networks make use of the Adam optimizer.

**Table 2:** The proposed network parameters.

Parameter	Value
Batch size	128
Epochs	30
Initial learning rate	0.001
Image input size	256 × 256 × 3
Loss function	Cross-entropy
Optimizer	Adam
Activation function	Rectified linear unit

TV is one of the most basic filter-based unsupervised feature selection approaches^27,28^. The variance of each feature in the features matrix is used to rank features. The variance along each dimension shows the dimension's representative power. As a result, TV can be utilized as a criterion for feature selection and extraction. It has already been used to select features from the face database for clustering^27^, as well as for text mining^28^. To determine which features to employ, this approach calculates the variance for each image patch. The TV algorithm searches the matrix for features that fulfill both the non-uniform feature distribution and the high feature frequency criterion. The process was implemented by calculating the variance of each feature, ${f}_{j}$, in the features matrix. TV is a variance score calculated using the following formula:

$$TV\left({f}_{j}\right)=Var\left({f}_{j}\right)=\frac{1}{N}\sum_{i=1}^{N}{\left({f}_{ij}-\overline{{f}_{j}}\right)}^{2}(i=1, 2, 3, \dots \dots , N;j=1, 2, 3, \dots .., M)$$

where $N and M$ are the features’ matrix dimensions which N represents the number of BC patches and M represents the number of features. The $\overline{{f}_{j}}$ is the mean of ${f}_{j}$. The discriminative feature receives a high Variance score (high TV).

**Figure 1:**
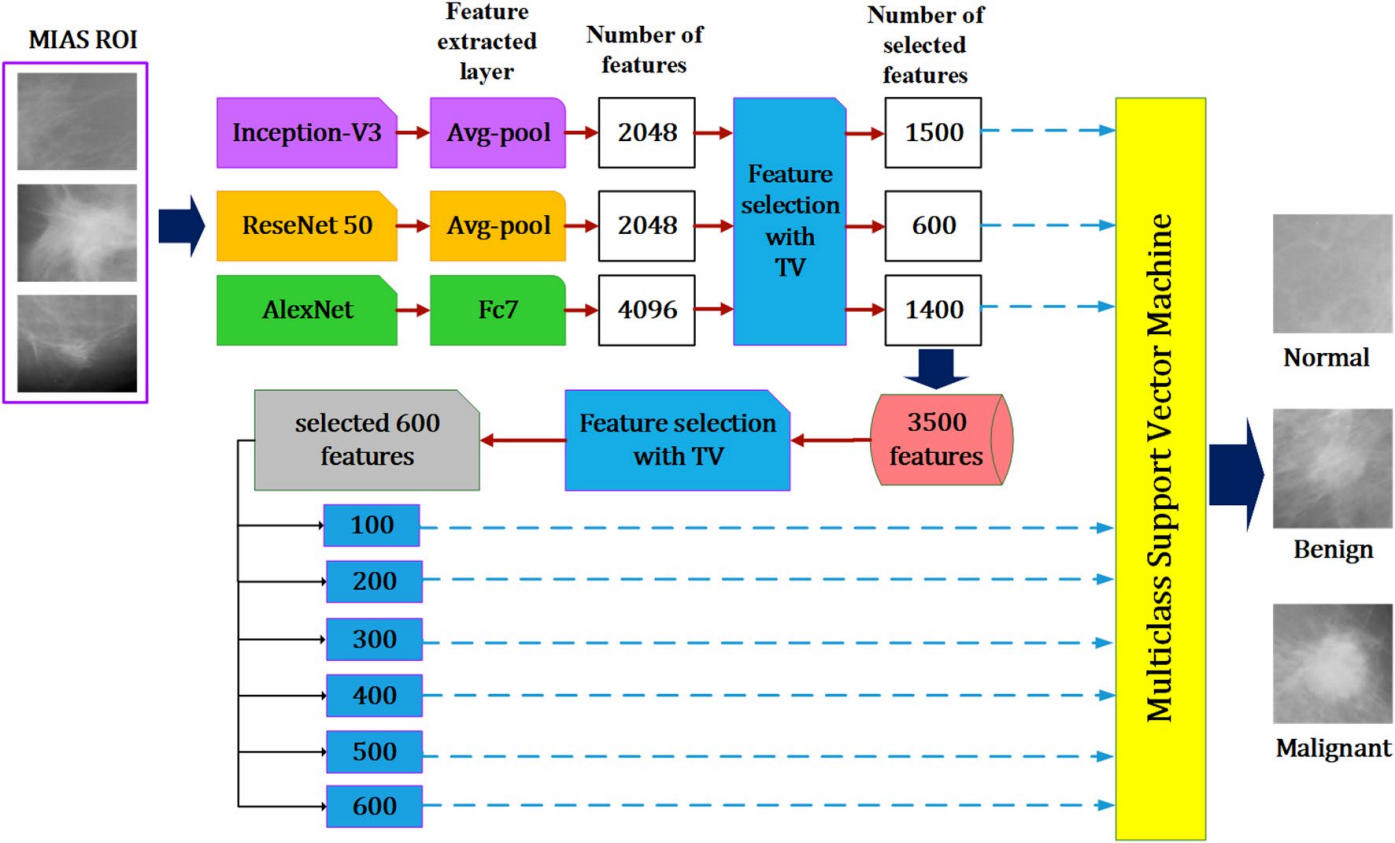
The concept of the suggested method.

The architecture of the suggested classification algorithm is shown in Fig. 1. The input images from the prepared dataset are scaled in the first stage to fit each pretrained network. From each input image, the Inception-V3 and ResNet50 networks each generate 2048 features on their global average pooling layers (Avg-pool). 4096 features are generated by AlexNet on its FC layer. Each CNN is submitted to the TV algorithm for feature selection. For Inception-V3, ResNet50, and AlexNet, TV generates 1500, 600, and 1400 features, respectively. The MSVM classifier received these features individually to evaluate the classification performance of each DL CNN algorithm. In the following stage, the 3500 total selected features that were gathered from the three DL CNN architectures were grouped into a single feature vector. The TV algorithm is used once more to further reduce the number of features. 600 features with the best prediction ability were found and split into 100 sub-features. The MSVM classifier received feature subsets of 100, 200, 300, 400, 500, and 600, respectively. Finally, the classification performance was assessed. As a result, the TV algorithm was used to first decrease a total of 8192 features to 3500 features, and then to further reduce them to 600 features. Based on the obtained classification performance and comparison to other published approaches, the 600-feature selection had the highest classification performance.

## Experimental setup

### Implementation

In our study, two stages are used to identify and categorize BC in the MIAS dataset. 416 mammography image patches are used at each stage to train suggested models and divide mammogram patches into three groups: normal, malignant, and benign. In each stage, this scenario was repeated for F, D, G, and a combination of them (All). In the first stage, features are extracted using the three individually pretrained DL CNNs (Inception-V3, ResNet50, and AlexNet), and reduced using the TV algorithm. For the classification task, the selected features feed MSVM. The proposed method was applied to a total of 3500 features that were chosen from the three pretrained CNNs in the second stage. 100 sub-features were created from the top 600 features with the best prediction capabilities. The MSVM classifier received inputs of 100, 200, 300, 400, 500, and 600 features. All models are trained using 70, 80, and 90% of the dataset.

### Evaluation metrics

To illustrate the suggested model's performance, the Receiver Operation Characteristics (ROC) curve and Confusion Matrix (CM) are used. The performance of CNN and the suggested networks are also assessed using the following metrics: Specificity, Recall, Precision, Accuracy, and F1- Score, as follows.

$$specificity=\frac{{T}_{N}}{{T}_{N}+{F}_{P}}$$

$$Recall=\frac{{T}_{P}}{{T}_{P}+{F}_{N}}$$

$$Precision=\frac{{T}_{P}}{{T}_{P}+{F}_{P}}$$

$$Classification \; Accuracy=\frac{No. \; of \; images \; correctly \; classified}{Total\; no. \;of\; images}=\frac{{T}_{P}+{T}_{N}}{{T}_{N}+{T}_{P}+{F}_{N}+{F}_{P}}$$

$$F1{\text{-}}Score=\frac{2 \times Precision \times Recall}{Precision + Recall}$$

where *T*_*N*_ and *T*_*P*_ are the sum of all true negative and true positive respectively. *F*_*N*_ and *F*_*P*_ are the sum of all false negative and false positive, respectively.

## Results and discussion

**Figure 2:**
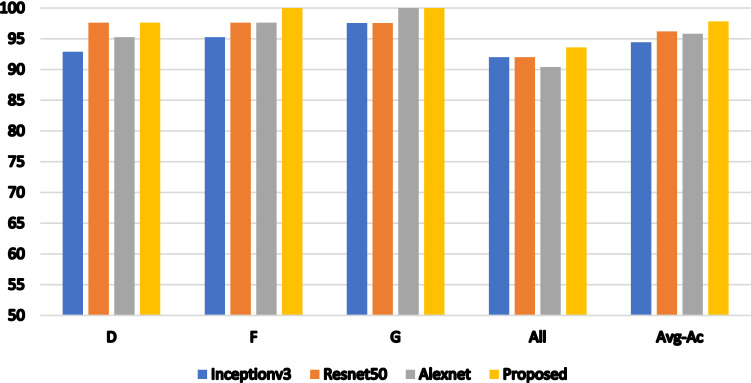
The accuracy comparison between CNNs models and the proposed one.

**Figure 3:**
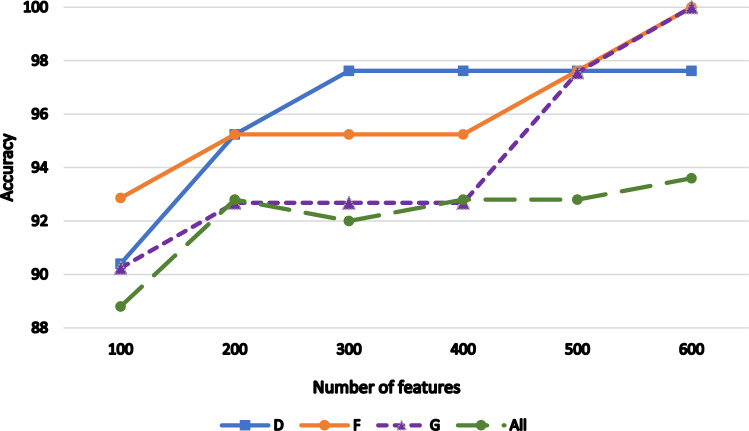
Accuracy of CNN-TV-SVM method with different sub-feature sets.

Table 3 and Fig. 2 reveal the findings of our experiment's initial stage. The table compares the CA of the individually pretrained Inception-V3, ResNet50, AlexNet DL CNN models with that of the suggested model with a 70% training rate. For F and G types, the proposed model obtained the optimal CA of 100%. Higher performance is also obtained for the other types (D, and All) compared to individually pretrained DL CNN models. Using the suggested model, an average CA of 97.81% is attained. Table 4 presents the results of the second stage with a 70% training rate. The table, which is also shown in Fig. 3, clarifies the impact of several selected features on the CA of the suggested model. It is obvious that, 600 features achieve the highest CA. As seen in Fig. 3, the performance rate quickly increased as the number of feature sets increased from 100 to 200. In each features’ subset, the rate increased slightly. As a result, instead of a total of 8192 features that were reduced to 3500 features in the first stage and then to 600 features in the second stage, the combined CNNs with the TV feature selection algorithm achieved the highest performance with only 600 features.

**Table. 3:** The classification accuracy of different pretrained networks and the proposed one.

Net	D %	F %	G %	All %	Avg_CA
Inceptionv3	92.86	95.24	97.56	92	94.42
ResNet50	97.62	97.62	97.56	92	96.2
AlexNet	95.24	97.62	100	90.40	95.82
Ours	97.62	100	100	93.6	97.81

**Table 4:** The CA of features subsets.

No. features	100	200	300	400	500	600
D	90.4	95.24	97.62	97.62	97.62	97.62
F	92.86	95.24	95.24	95.24	97.62	100
G	90.24	92.68	92.68	92.68	97.56	100
All	88.8	92.8	92	92.8	92.8	93.6

**Figure 4:**
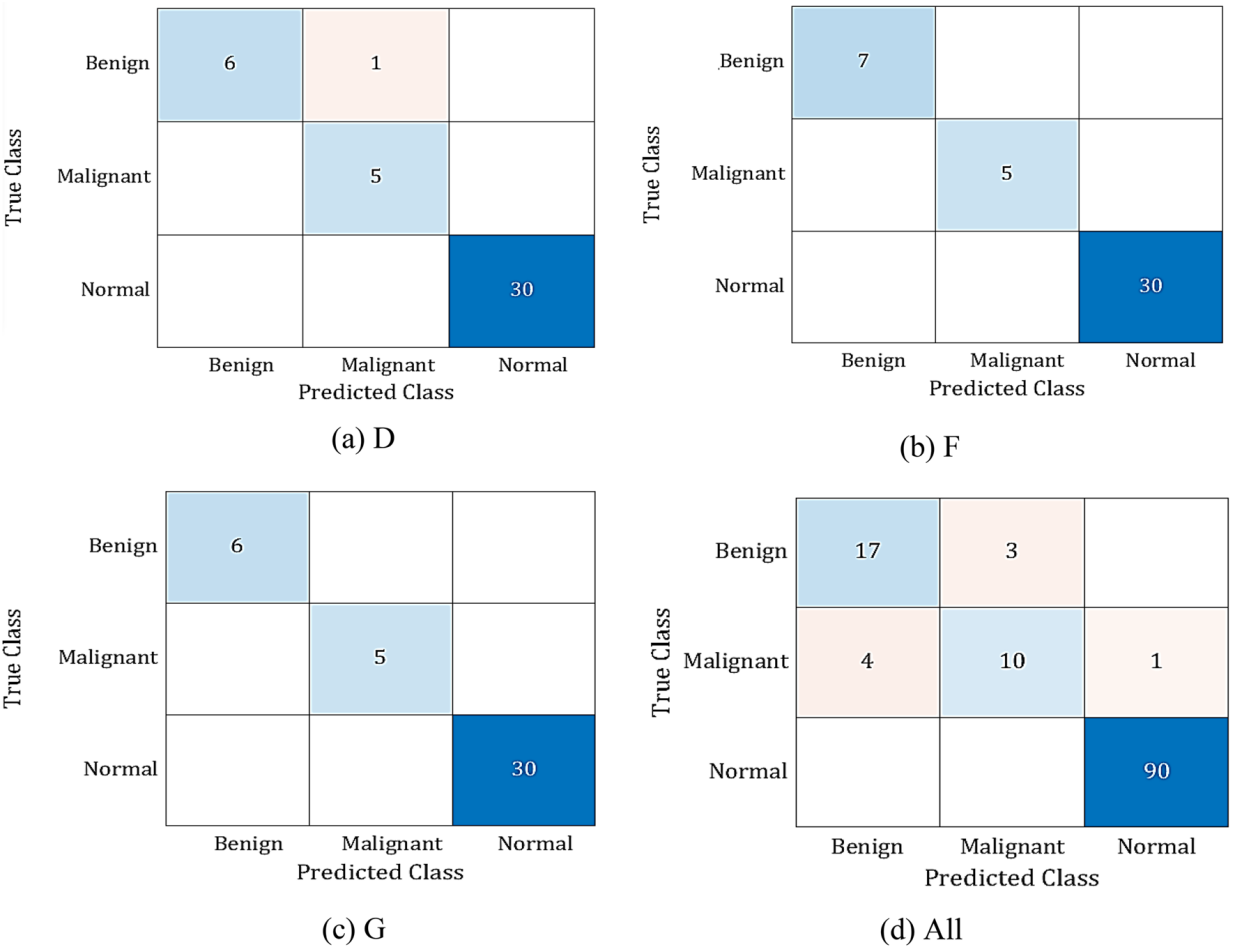
Confusion matrices of the proposed method.

**Figure 5:**
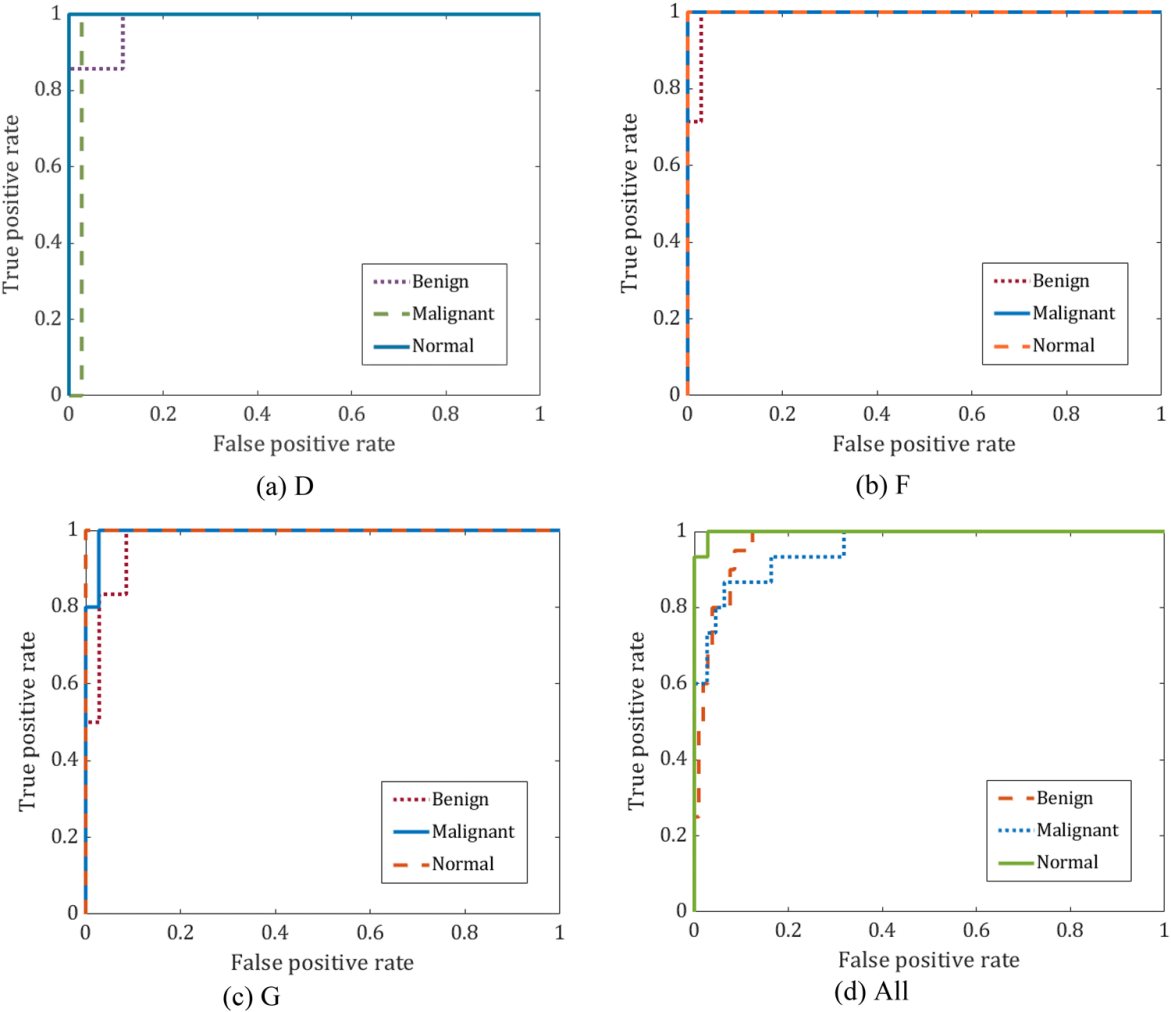
ROC curves of proposed mothed.

Specificity, recall, precision, accuracy, F1-score, and AUC are the key parameters used to assess the effectiveness of the suggested method. The average performance of the proposed model for D, F, and G breast tissue types, and All of them together are presented in Table 5. The proposed model, according to the table, performed best for the F and G types and somewhat less for the remaining types, where an acceptable average performance was reached. The suggested model is depicted as a CM and ROC in Figs. 4 and 5, respectively. 42 mammography patches of the G and F types are examined, and each patch receives the appropriate classification. Out of 41 patches of D type, only 1 is incorrectly classed. 8 of the 117 patches of the All type have been classified wrongly. The ROC curves in Fig. 5 show that the F tissue type achieves the best classifier performance. For other types, though, a good performance was attained.

**Table 5:** Average performance of the proposed method for different breast tissue types.

	Recall	Specificity	precision	F1-score	AUC
D	95.24	99.1	94.44	94.41	98.56
F	100	100	100	100	99.73
G	100	100	100	100	99.02
All	83.89	96.87	85.6	84.6	97.54
Average	94.78	99	95	94.75	98.72

**Figure 6:**
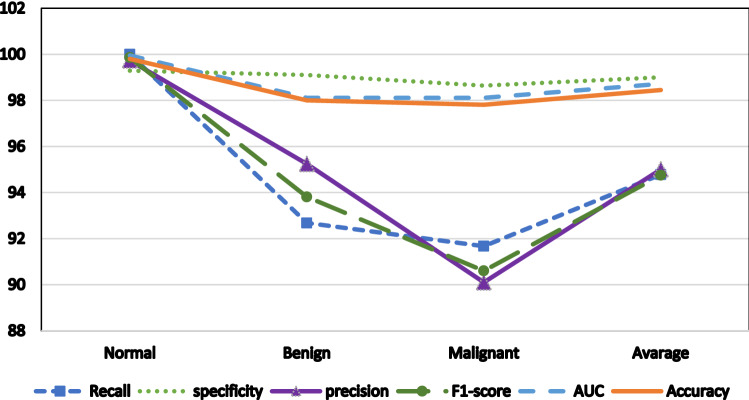
The proposed method average performance of all tissue type.

Table 6 and Fig. 6 provide descriptions of the average individual performance of the proposed method for all breast tissue types "All" for various classes (i.e., normal, malignant, and benign). The performance rate decreases from the normal to the benign to the malignant class, as seen in the figure. The normal class achieves the maximum performance, whereas the malignant class achieves the lowest performance.

**Table 6:** Average individual performance of the proposed method for different classes.

	Recall	Specificity	Precision	F1-score	AUC	Accuracy
Normal	100	99.29	99.73	99.86	99.95	99.8
Benign	92.68	99.1	95.24	93.81	98.11	98
Malignant	91.67	98.64	90.1	90.6	98.11	97.81

Finally, different training rates—70%, 80%, and 90% of our provided dataset—were used to train the individual DL CNNs and the suggested model. Table 7 displays the performance outcomes of the DL CNN models in comparison to the suggested model. The table clearly shows that the suggested model beats existing CNN models in CA, achieving an optimal CA of 100% at a 90% training rate.

**Table 7:** The CA of the individual DL CNN models and the proposed one.

Training rate	70%	80%	90%
Inception-v3 + TV	92	92.77	95.24
ResNet50 + TV	92	94	97.62
AlexNet + TV	90.40	92.77	97.62
Ours	93.6	95.2	100

Additionally, as shown in Table 8, the proposed strategy has been compared with other current state-of-the-art researches that make use of MIAS mammography dataset. In the table for 3-class cases in the term of CA, the performance values of each study are presented. Results indicate that the suggested model outperformed other models.

**Table 8:** The CA % of the proposed method and other existing methods.

Reference	Year	Method	CA%	Training ratio
^10^	2006	Co-ocurance + Bayesian neural networks	86.48	–
^11^	2017	RF	80	–
		RF-ELM	95	
^18^	2017	CNN	68	70%
^33^	2017	CNN + SVM	92.85	50%
^14^	2018	PB- DCT	92	50%
^30^	2018	Gestalt psychology	92	90%
^15^	2019	Hough transform + SVM	94	50%
^16^	2019	SVM	0.94	90%
^17^	2019	GMM + SVM	90	33%
^13^	2019	DL + SVM	96.9	70%
		DL + KNN	93.8	
		DL + LDA	89.7	
		DL + DT	88.7	
^35^	2019	MSER detector and features matching	96.47	-
^32^	2019	Meta-heuristic algorithm EML	78.57	-
^31^	2019	Contourlet + FOA + NB	95.93	70%
^23^	2019	DenseNet201	92.73	80%
^24^	2020	AlexNet	90.3	75%
		Googlenet	92.2	
^12^	2020	Optimized KELM	97.49	80%
^38^	2020	DWT + GLCM + DBN	91.5	-
^36^	2020	FC-CSO-CRNN	90.598	-
		Integration of CNN and RNN		
^37^	2021	BDR-CNN-GCN	96.1	55%
^19^	2021	SVM-RBF	71.43	80%
		ELM	76.09	
		PSO-ELM	86.03	
		CS-ELM	93.17	
		ICS-ELM	95.96	
^21^	2021	CNN	93.75	-
^41^	2022	ResNet50	89.5	90%
		Nasnet-Mobile	70	
^42^	2022	TTCNN	96.57	60%
^39^	2022	ADL-BCD	93.17	70%
			93.58	80%
			96.07	90%
Ours		CNNs + TV + MSVM	97.81	70%
			98	80%
			100	90%

### Ablation analysis

To examine the efficiency of the key elements (i.e., CNN networks and TV) in our proposed architecture, we conduct ablation studies, and the numerical outcomes are shown in Table 9. Only the studied component is eliminated from the proposed system during each ablation research, while the others remain. The impact of eliminating each of the three pretrained networks is investigated. In each ablation trial, two CNNs are employed, and 600 features are chosen using the TV model and supplied to the MSVM for classification. In comparison to the proposed network, the CA is reduced without (W/o) ResNet50, Inception-V3, or AlexNet, and the highest reduction occurs without ResNet50, as shown in Table 9. The effect of the TV feature selection model is also examined. In this investigation, the three CNNs' 8192 extracted features are all sent to the MSVM Classifier, which performs the classification operation. As noticed in the table, without the TV, the worst CA is reached. The best CA is realized only when the proposed network is utilized.

**Table 9:** Ablation study of key components (CNNs and TV) in our method.

Methods	70% training rate	80% training rate	90% training rate
W/o ResNet50	91.2%	92.77%	93%
W/o Inception-V3	92%	92.77%	95.24%
W/o AlexNet	92%	92.77%	95.24%
W/o TV	91.2%	91.6%	93%
Ours	93.6%	95.2%	100%

## Conclusion

This paper proposes and tests a new automated BC detection and classification algorithm with the fewest possible features. The Inception-V3, ResNet50, and AlexNet CNN models, three of the most popular pretrained architectures, provided the effective DL features used in this model. In the two stages of the experiment, the TV algorithm is applied twice for the selection of robust high-ranking features. Using the TV algorithm, features are chosen from each distinct DL CNN model in the initial stage and provided to the MSVM classifier independently. 3500 robust features were left out of the original 8192 features. These features were subjected to the TV algorithm once more, which reduced them to 600 weighted features that influence classification performance. MSVM was utilized to classify the first 100, 200, 300, 400, 500, and 600 features with the highest feature weight. The newly proposed hybrid technique, which combines CNNs + TV + MSVM, obtained 97.81% for training on 70% of the data, 98% for training on 80% of the data, and meets the ideal value of 100% for training on 90% of the data. When compared with separate DL CNN models, i.e., InceptionV3, ResNet 50, and AlexNet, as well as other studies in the literature, the suggested hybrid technique achieves the highest performance for BC diagnosis. The importance of the proposed network's key parameters is highlighted using the ablation analysis.

## Data Availability

The datasets analyzed during the current study are *publicly available* in the (mammographic image analysis homepage) repository, (https://www.mammoimage.org/databases/).
